# AIDS Patient Death Caused by Novel *Cryptococcus neoformans* × *C*. *gattii* Hybrid 

**DOI:** 10.3201/eid1407.080122

**Published:** 2008-07

**Authors:** Marjan Bovers, Ferry Hagen, Eiko E. Kuramae, Hans L. Hoogveld, Françoise Dromer, Guy St-Germain, Teun Boekhout

**Affiliations:** * Centraalbureau voor Schimmelcultures–Fungal Biodiversity Centre, Utrecht, the Netherlands; † University Medical Centre Utrecht, Utrecht; ‡ Netherlands Institute of Ecology, Nieuwersluis, the Netherlands; § Institut Pasteur, Paris, France; ¶ Laboratoire Santé Publique Quebec, Sainte-Anne-de-Bellevue, Quebec, Canada; 1 These authors contributed equally to this article.; 2 Current affiliation: Netherlands Commission on Genetic Modification, Bilthoven, the Netherlands.

**Keywords:** Cryptococcus neoformans, Cryptococcus gattii, hybrids, serotype AB, HIV, AIDS, dispatch

## Abstract

Interspecies hybrids of *Cryptococcus neoformans* and *C*. *gattii* have only recently been reported. We describe a novel *C. neoformans* × *C. gattii* hybrid strain (serotype AB) that was previously described as *C. gattii* and that caused a lethal infection in an AIDS patient from Canada.

*Cryptococcus neoformans* and *C*. *gattii* are pathogenic yeasts that may cause meningoencephalitis. *C*. *neoformans* primarily infects immunocompromised patients and occurs worldwide, whereas *C*. *gattii* primarily mainly infects otherwise healthy persons and has been thought to occur in subtropical regions (*1*–*3*). However, the recent outbreak of infection with *C*. *gattii* on Vancouver Island, British Columbia, Canada (*4*), expansion of this outbreak to mainland Canada and the Pacific Northwest region of the United States (*5*), and identification of *C*. *gattii* isolates in Europe (*6*) show that *C*. *gattii* can also occur in temperate climates. Molecular techniques can distinguish 7 haploid genotypic groups within *C*. *neoformans* and *C*. *gattii* (*7*–*9*; F. Hagen and T. Boekhout, unpub. data).

Recently, 3 serotype BD *C*. *neoformans* var. *neoformans* × *C*. *gattii* hybrids were isolated from 2 HIV-negative patients in the Netherlands (*10*). We describe a novel *C*. *neoformans* var. *grubii* serotype A × *C*. *gattii* serotype B hybrid that was isolated from an HIV-positive person.

## The Study

Strain CBS10496 was isolated from a 31-year-old AIDS patient from Montreal, Quebec, Canada, who had traveled to Mexico ≈15 months before cryptococcosis was diagnosed. The patient died despite extensive antifungal treatment with ketoconazole and amphotericin B (*11*). CBS10496 has been identified as *C*. *gattii* serotype B (cited as *C*. *neoformans* var. *gattii*) (*11*). Reference isolates used in this study are listed in the Table.

**Table:** *Cryptococcus* spp. strains used in this study*

Strain†	Mating type‡/serotype	AFLP/M13 genotype§	Source	Country
CBS9172	aA	AFLP1/VNI	Soil sample from garden of patient with neighboring bird colonies	Italy
CBS8710 (H99)	αA	AFLP1/VNI	Patient with Hodgkin disease	United States
CBS1622	αB	AFLP4/VGI	Tumor	France
CBS6992	αB	AFLP4/VGI	Human	Unknown
E566	aB	AFLP4/VGI	*Eucalyptus camaldulensis*	Australia
CBS10510 (WM276)	αB	AFLP4/VGI	Debris of *E*. *tereticornis*	Australia
CBS10488 (AMC770616)	αBaD	AFLP8/NA	Human CSF	The Netherlands
CBS10489 (AMC2010404)	αBaD	AFLP8/NA	Human CSF	The Netherlands
CBS10490 (AMC2011225)	αBaD	AFLP8/NA	Human CSF	The Netherlands
CBS10496 (LSPQ#308)	αA–B	AFLP9/NA	Blood of an HIV-positive patient	Canada

*AFLP, amplified fragment length polymorphism; CBS, Fungal Biodiversity Centre; AMC, Netherlands Reference Laboratory for Bacterial Meningitis, Academic Medical Center, Amsterdam, the Netherlands; NA, not applicable; CSF, cerebrospinal fluid. †Origin and genetic composition of the strains are indicated. ‡ –, mating type unknown. §AFLP fingerprint genotype (*7*), followed by corresponding M13 PCR fingerprint genotype (*8*).

The ploidy of CBS10496 was determined by using flow cytometry (*10*) with the sequenced haploid strains CBS8710 and CBS10510 as references. Nuclei were visualized after staining with 4′,6-diamidino-2-phenylindole (*10*). Coloration of colonies grown on canavanine-glycine-bromthymol blue (CGB) medium (*12*) was determined after incubation at 24°C for 6 and 15 days. The serotype of CBS10496 was determined by using the CryptoCheck serotyping kit (Iatron Laboratories, Tokyo, Japan).

Ten colonies of CBS10496 were used for DNA extraction (*10*). DNA of these colonies was used for amplified fragment length polymorphism (AFLP) analysis (*7*). The partial sequence of 6 nuclear regions was determined for reference isolates CBS10488–CBS10490, CBS1622, CBS6992, and the putative hybrid isolate CBS10496. Selected nuclear regions were those for internal transcribed spacer (ITS) region, intergenic spacer region, laccase (*CNLAC1*), 2 RNA polymerase II subunits (*RPB1* and *RPB2*), and translation elongation factor 1α (*TEF1*α) (*9*,*10*). Mating types and serotype were determined as described (*10*,*13*).

**Figure 1:**
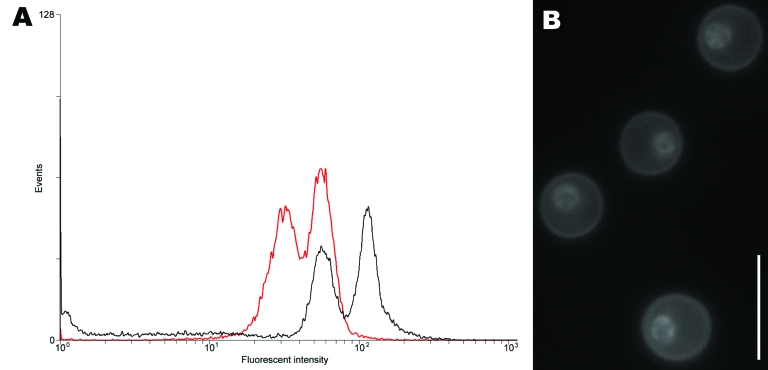
A) Determination of ploidy of the novel *Cryptococcus neoformans* × *C*. *gattii* serotype AB hybrid isolate CBS10496 by flow cytometry. The first peak corresponds to the G1 phase; the second peak corresponds to the G2 phase. Haploid reference strain CBS10510 is shown by the red line; CBS10496 is shown by the black line. The G1 peak of CBS10496 coincided with the G2 peak of strain CBS10510, which indicated that strain CBS10496 has approximately twice the amount of DNA than CBS10510. B) Nuclear staining of isolate CBS10496 with 4′,6-diamidino-2-phenylindole, showing that cells are monokaryotic. Scale bar = 10 μm.

DNA content of CBS10496 was compared with that of CBS8710 and CBS10510. The G1 peak of reference strains was located at positions 31.6 (CBS8710) and 31.1 (CBS10510), and the G2 peak was located at positions 65.8 (CBS8710) and 56.4 (CBS10510). The G1 peak of CBS10496 was located at position 57.5, and the G2 peak was located at position 115.7. Thus, the G1 peak of CBS10496 coincided with the G2 peak of the haploid strains (Figure 1, panel **A**), which indicates that CBS10496 has ≈2× more DNA than haploid strains. We concluded that CBS10496 is diploid or aneuploid. Staining with 4′,6-diamidino-2-phenylindole showed that cells of CBS10496 were monokaryotic (Figure 1, panel **B**).

Reaction of CBS10496 on CGB medium was negative, which corresponds to *C*. *neoformans* (*12*). The CryptoCheck serotyping kit serum factors 5 (corresponding to serotype B) and 7 (corresponding to serotype A) agglutinated, which indicated that CBS10496 is a serotype AB strain.

**Figure 2:**
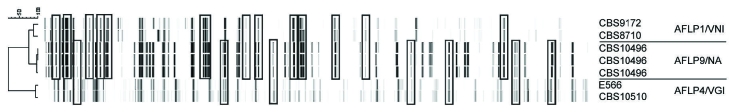
Amplified fragment length polymorphism (AFLP) fingerprint of 3 colonies of the novel *Cryptococcus neoformans* × *C*. *gattii* hybrid serotype AB isolate CBS10496 and 4 reference strains. CBS9172 and CBS8710 are *C. neoformans* var. *grubii* (AFLP1/VNI) strains; E566 and CBS10510 are *C. gattii* (AFLP4/VGI) strains. Rectangles indicate AFLP fragments characteristic for AFLP1/VNI or AFLP4/VGI and present in isolate CBS10496.

The AFLP fingerprint obtained by analysis of colonies of CBS10496 did not match any of the previously defined AFLP genotypes. The fingerprint of CBS10496 was compared with AFLP fingerprints of reference strains CBS8710 and CBS9172, which are AFLP1/VNI, and E566 and CBS10510, which are AFLP4/VGI. The AFLP fingerprint of CBS10496 contained fragments characteristic of AFLP1/VNI and AFLP4/VGI (Figure 2), which indicated that genetic material from these 2 genotypes was present in this isolate.

Two alleles representing AFLP1/VNI and AFLP4/VGI were found when fragments of *RPB1*, *RPB2*, *CNLAC1*, and intergenic spacer region of CBS10496 were cloned and sequenced. However, after 30 clones were sequenced, only 1 allele was obtained for *TEF1*α, i.e., AFLP4/VGI, and ITS, i.e., AFLP1/VNI. Our results indicate that genetic material from AFLP1/VNI and AFLP4/VGI was present in CBS10496, although only 1 allele was obtained for *TEF1*α and ITS. All sequences were submitted to GenBank (accession nos. DQ286656–DQ286676 and EF102027–EF102072).

Amplification of CBS10496 in a PCR with the *MAT*α and the *MAT*α serotype A–specific primer pair resulted in an amplicon. When *MAT*a and the *MAT*a serotype A–specific PCRs were conducted, no amplicon was obtained. These findings indicate that CBS10496 has a *MAT*α serotype A background. All reference strains yielded amplicons with the expected primer pairs. In addition, CBS10510, a *MAT*α serotype B strain, was amplified with the *MAT*α specific primer pair, and E566, a *MAT*a serotype B strain, yielded an amplicon with the *MAT*a-specific PCR. These results indicate that a *C*. *gattii* and a *MAT*α serotype A background are present in CBS10496. Because the mating type of the *C*. *gattii* background within CBS10496 was unknown, 30 *MAT*α clones of CBS10496 were sequenced to determine whether a *MAT*α serotype B allele could be identified. However, all clones were *MAT*α serotype A; no *MAT*α serotype B clones were found.

## Conclusions

Our results indicated that CBS10496 is a monokaryotic, diploid, or aneuploid strain with the novel AB serotype. AFLP and sequence analysis showed that the isolate contained fragments of *C*. *neoformans* var. *grubii* (AFLP1/VNI) and *C*. *gattii* (AFLP4/VGI). We conclude that this isolate is a novel aneuploid hybrid of *C*. *neoformans* var. *grubii* (serotype A, AFLP1/VNI) and *C*. *gattii* (serotype B, AFLP4/VGI).

CBS10496 had been identified as *C*. *gattii* on the basis of a weak positive reaction on CGB medium (*11*). Our results indicated that CBS10496 was negative on CGB medium. Although a negative response on CGB medium has been shown for other *C*. *neoformans* × *C*. *gattii* hybrids (*10*,*14*), weak and delayed positive reactions on CGB medium may occur in *C*. *neoformans* × *C*. *gattii* hybrid isolates (*10*,*14*). CBS10496 was previously identified as a serotype B strain (*11*). Inconsistent serotyping results have been reported for other hybrids (*10*,*15*) and may result from differences in specificity and potency among different batches of factor serum. All *C*. *neoformans* × *C*. *gattii* hybrids discovered have originated from clinical sources (*13*; F. Hagen and T. Boekhout, unpub. data).

We expected that CBS10496 would have 2 mating-type loci. However, only a serotype A *MAT*α background was observed. Although an amplicon was obtained with *C*. *gattii*–specific mating-type primers, the *C*. *gattii* background could not be linked to a mating type. We hypothesize that the serotype AB *C*. *neoformans* × *C*. *gattii* hybrid CBS10496 was formed by mating of a *MAT*a serotype B strain with a *MAT*α serotype A strain and subsequent loss of the *MAT*a serotype B allele. Detection of single ITS and *TEF1*α alleles in CBS10496 further supports our findings because it indicates that other alleles were also lost. Loss of genetic material has been observed in other hybrids, such as serotype AD and BD hybrids (*14*), and seems to be a normal process in cryptococcal hybrids.

Our results show that the *C*. *gattii* parent of the serotype AB hybrid belongs to the AFLP4/VGI genotype, as was the case for serotype BD hybrids (*10*). The *C*. *gattii* parental sequence of all known serotype BD *C*. *neoformans* × *C*. *gattii* hybrid isolates was identical to sequences of AFLP4/VGI strains CBS1622 and CBS6992 in all regions studied (*9*). Detection of 1 specific *C*. *gattii*–AFLP4/VGI subgroup in all isolated *C*. *neoformans* × *C*. *gattii* hybrids may indicate that this subgroup preferentially forms interspecies hybrids.
